# Cushing’s Syndrome Effects on the Thyroid

**DOI:** 10.3390/ijms22063131

**Published:** 2021-03-19

**Authors:** Rosa Maria Paragliola, Andrea Corsello, Giampaolo Papi, Alfredo Pontecorvi, Salvatore Maria Corsello

**Affiliations:** Department of Translational Medicine and Surgery, Unit of Endocrinology, Università Cattolica del Sacro Cuore-Fondazione Policlinico “Gemelli” IRCCS, Largo Gemelli 8, I-00168 Rome, Italy; rosamariaparagliola@gmail.com (R.M.P.); papigiampaolo@hotmail.com (G.P.); alfredo.pontecorvi@unicatt.it (A.P.); corsello.sm@meridiaroma.it (S.M.C.)

**Keywords:** Cushing’s syndrome, hypothalamus–pituitary–thyroid axis, thyroid function tests

## Abstract

The most known effects of endogenous Cushing’s syndrome are the phenotypic changes and metabolic consequences. However, hypercortisolism can exert important effects on other endocrine axes. The hypothalamus–pituitary–thyroid axis activity can be impaired by the inappropriate cortisol secretion, which determinates the clinical and biochemical features of the “central hypothyroidism”. These findings have been confirmed by several clinical studies, which also showed that the cure of hypercortisolism can determine the recovery of normal hypothalamus–pituitary–thyroid axis activity. During active Cushing’s syndrome, the “immunological tolerance” guaranteed by the hypercortisolism can mask, in predisposed patients, the development of autoimmune thyroid diseases, which increases in prevalence after the resolution of hypercortisolism. However, the immunological mechanism is not the only factor that contributes to this phenomenon, which probably includes also deiodinase-impaired activity. Cushing’s syndrome can also have an indirect impact on thyroid function, considering that some drugs used for the medical control of hypercortisolism are associated with alterations in the thyroid function test. These considerations suggest the utility to check the thyroid function in Cushing’s syndrome patients, both during the active disease and after its remission.

## 1. Introduction

Endogenous Cushing’s syndrome (CS) is caused by a prolonged exposure to high endogenous circulating levels of cortisol. In about 70% of cases, CS is caused by an adrenocorticotropic hormone (ACTH)-producing pituitary tumor (Cushing’s disease, CD), while a primary adrenal hyperfunction (ACTH-independent CS, ACS) and ectopic ACTH-secreting neuroendocrine tumor (EAS) account for the remaining 30% of cases [1]. Hypercortisolism represents a challenging condition, because if not promptly diagnosed and treated, it leads to increased morbidity and mortality. Hypercortisolism is characterized by specific clinical features (moon face, facial plethora, striae rubrae, and supraclavicular and dorsal fat pads), as well as metabolic complications such as visceral obesity, hypertension, diabetes, dyslipidemia, osteoporosis [2], and detrimental effects on the brain, including hippocampal damage and memory impairment [3]. Moreover, the inappropriate cortisol secretion impairs the function of other endocrine axes. In both men and women, hypogonadotrophic hypogonadism is common, and it correlates with the severity of hypercortisolism [4]. Furthermore, the glucocorticoid (GC) excess inhibits growth hormone (GH) secretion, probably by altering the hypothalamic somatostatin tone [5], and it reduces the GH secretion during sleep and the GH response to dynamic stimulation tests [6]. Glucocorticod excess also acts on the hypothalamus–pituitary–thyroid (HPT) axis, reducing the secretion and the activity of thyroid hormones by different mechanisms. Both thyrotropin-releasing hormone (TRH) and thyroid-stimulating hormone (TSH) release, as well as the deiodinase activity, have been shown to be altered by hypercortisolism. Moreover, some of the drugs used in the treatment of CS can interfere with thyroid function, and the resolution of hypercortisolism has been associated with an increased occurrence of thyroid diseases. However, the clinical significance of the dynamic changes in HPT axis activity during hypercortisolism and after the resolution of the inappropriate cortisol secretion is still debated. From a clinical point of view, the monitoring of thyroid function tests in CS patients can be useful. This article will review the dynamic changes of the hypothalamus–pituitary–thyroid axis during CS and after its resolution, with an emphasis on the effects induced by the drugs employed for its treatment.

## 2. Effects of Glucocorticoids on Hypothalamus–Pituitary–Thyroid Axis

Normal thyroid function is the result of the complex and well-coordinated activity of the HPT axis, which is influenced by the interaction with external factors and regulated by a negative feedback loop mechanism. Pituitary TSH secretion, which in turn is dependent on hypothalamic TRH release, represents the main regulator of thyroid development and function. In physiological conditions, the steady-state concentration of TSH is determined by the balance of the stimulatory action of TRH and the negative feedback of thyroid hormones, acting on both TRH and TSH release [7], as well as by other regulators originating from the central nervous system, peripheral tissues, and local pituitary mechanisms [8]. In animal models, T3 downregulates the expression of TRH mRNA in the hypophysiotropic neurons of the paraventricular nucleus (PVN) [9], and the neuronal concentration of T3 in the PVN depends on the type 2 deiodinase (D2)-mediated conversion of T4 in tanycytes, on the presence of T3 transporters, and on the activity of neuronal type 3 deiodinase (D3) that inactivates T3 [10].

Hypothalamus–pituitary–adrenal axis function can influence the normal HPT activity. In rats, neurons showing immunoreactivity for TRH located in the dorsal hypothalamus and in the anterior periventricular hypothalamic nucleus revealed strong immunoreactivity also for GC receptors (GR) [11]. Studies evaluating GR expression in human surgically removed pituitary adenomas using a polyclonal GR antibody, and it also revealed nuclear and/or cytoplasmic GR immunoreactivity in many non-tumorous corticotrophs and other adenohypophysial cell types [12]. GR immunoreactivity was also demonstrated in many GH, prolactin (PRL), ACTH, TSH, follicle-stimulating hormone (FSH), luteinizing hormone (LH) α-subunit producing adenomas, null cell adenomas, and oncocytomas. Interestingly, suppressed non-tumorous corticotrophs in CD exhibited only weak or no GR immunopositivity, indicating a GR downregulation related to the inappropriate cortisol secretion [12].

According to the evidence that confirms the expression of GR in thyrotrophs and TRH-secreting neurons, chronic GC exposure has been demonstrated to decrease *Trh* expression in the PVN and TSH serum concentrations in rat and in human [13,14], as well as to increase the Neuropeptide Y (NPY) expression, which in turn contributes to the GC-inhibitory effect on *Trh* mRNA levels in the PVN. Postmortem studies on subjects in therapy with synthetic GC showed the reduced expression of *Trh* in the PVN [14]. This phenomenon has been reported also for patients with major depression, in which a stress-related endogenous hypercortisolism can be supposed [15].

In vitro studies suggest that GC affects TSH secretion by exerting direct inhibitory effects on the pituitary gland [16]. Subsequent studies have demonstrated that annexin 1 (lipocortin 1) produced by the pituitary folliculostellate cell acts as a paracrine mediator of acute regulatory effects of glucocorticoids, both on TSH secretion and on ACTH, PRL, and LH secretion [17,18]. The role of somatostatin (SST) in the GC-mediated TSH inhibition has been reported. Somatostatin directly inhibits TSH secretion by activating the type 2 SST receptors (SSTR2) and type 5 SST receptors (SSTR5) expressed on the pituitary thyrotropes [19]. Glucocorticoids rapidly increase hypothalamic SST mRNA and SST release in animal models, amplifying the effects on TSH inhibition [20].

Furthermore, in humans, hypercortisolism has been demonstrated to reduce TSH secretion and TSH pulse amplitude. In CS patients, the mean 24-h TSH levels are lower than in controls with a lower mean 24-h TSH pulse amplitude. The nocturnal TSH surge is also decreased, due to the loss of the usual nocturnal increase of TSH reported in healthy people [21]. An inverse relationship between night serum cortisol and TSH in CS patients has been reported [22], and a recent study proposed the use of the “night–day TSH ratio (2300–2400 h to 0800–0900 h)” ≤ 1 as diagnostic criterion for the differential diagnosis between CS and depression [sensitivity 90.9% (95% confidence interval), specificity 95.0% (95% confidence interval)] [23]. TSH response after TRH stimulation is impaired in endogenous hypercortisolism, in which the increase of TSH after TRH stimulation is significantly lower in CS patients than in healthy subjects [24]. These findings have been confirmed also by other authors, and no differences were found neither in the pattern of TSH secretion nor in the TSH response to TRH between patients with ACTH-dependent and those with ACTH-independent CS [22].

Therefore, hypercortisolism can be associated to the biochemical features characteristic of the “central hypothyroidism”. It might be speculated that in some CD patients, central hypothyroidism can be related to the “mass effect” exerted by the pituitary adenoma. However, it is well known that CD is often caused by microadenomas that, unlike pituitary macroadenomas, are not commonly associated with hypopituitarism. Furthermore, the higher prevalence of central hypothyroidism in ACTH-secreting microadenomas compared with microprolactinomas and non-functioning microadenomas has been reported [25], confirming the role of hypercortisolism in the genesis of central hypothyroidism.

Endogenous hypercortisolism also reduces thyroid hormone action on peripheral tissues by other mechanisms. In CS patients, reduced T4, T3, and FT3 and increased reverse T3 levels have been reported [22,24]. The decreased T3:T4 often reported can be due to GC-mediated inhibition of peripheral deiodination [26]. Consistent with this, after curative surgery, T3 levels increased disproportionately to T4 and FT4, reflecting the possible recovery of the type 1 and type 2 deiodinase activity, which converts T4 to T3, after the resolution of hypercortisolism [27]. Moreover, hypercortisolism is associated to a reduction in thyroxin biding globulin (TBG) [24], which contributes to the reduced T3 and T4 concentration.

**Figure 1:**
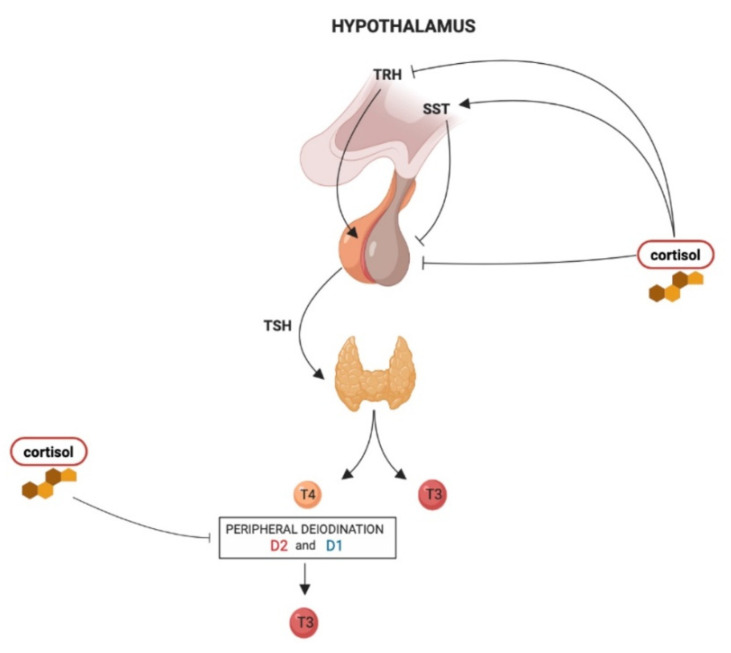
The influence of hypercortisolism on the hypothalamus–pituitary–thyroid (HPT) axis regulation: thyrotropin-releasing hormone (TRH) derived from the parvocellular neurons of the hypothalamic paraventricular nucleus (PVN), stimulates TSH synthesis and secretion from thyrotroph cells located in the anterior pituitary gland. In turn, TSH regulates thyroid hormone production and release via binding to the TSH receptor in thyroid follicular cells. The thyroid gland secretes T4 and about 20% of circulating T3. About 80% of circulating T3 is derived from the peripheral deiodination of T4 that is mediated by D2 (mainly) and D1. At the hypothalamic level, chronic hypercortisolism can reduce *Trh* expression in the PVN and increase somatostatin (SST) release, which in turn inhibits TSH release. At the pituitary level, hypercortisolism directly inhibits TSH secretion. Hypercortisolism also inhibits the peripheral deiodination of thyroid hormones with a consequent decrease in T3:T4 ratio.

The effects of hypercortisolism on the HPT axis are summarized in Figure 1.

## 3. Thyroid Function Dynamic Changes in Cushing’s Syndrome

The dynamic changes of thyroid function and the recovery time of HPT axis normal activity have been evaluated by different clinical studies. A prospective study performed on 27 patients with pituitary (16 patients) and adrenal (11 patients) CS showed reduced TSH secretion and TSH pulse mass during active disease, independently of hypercortisolism etiology [28]. Interestingly, FT4 levels were decreased only in pituitary-dependent hypercortisolism compared with controls. However, in this study, the cortisol production rate was higher in patients with CD than in patients with an adrenal adenoma, and total cortisol production was negatively correlated with TSH production and with FT4 concentration. Therefore, the more severe degree of hypercortisolism in CD in this specific group of patients could explain the specific correlation between CD and hypothyroxinemia. In the seven post-surgical “cured” patients, a normal TSH rhythm with elevated basal TSH release has been found [28]. The reduction of TSH levels during the active disease has been confirmed by other prospective studies, involving similar groups of patients [29,30], as well as the return to normal thyroid function after the cure of hypercortisolism [30]. On the contrary, other authors showed similar serum FT3 and FT4 levels in CS patients and controls, both during the active disease phase and after successful treatment [30].

Xiang and et al. evaluated the thyroid function in a group of 118 CS patients before and after surgery, including the evaluation of anti-thyroid autoantibodies in CD. Results showed that TSH, T3, and FT3 levels were below the reference range in 31%, 69%, and 44% of the 118 CS patients respectively, and they returned to normal within one year after surgery in most cases. TSH, T3, and FT3 were negatively correlated with serum cortisol levels, both before and after surgery, while no significant changes in anti-thyroid autoantibodies were observed [31].

A recent study retrospectively evaluated the thyroid function in two groups of patients affected by CS (cohort 1, 68 patients and cohort 2, 55 patients, who performed follow-up in different years), before and after successful surgery [27]. Data demonstrated that 53% of patients in cohort 1 had central hypothyroidism (low FT4 levels with inappropriately low TSH), while in cohort 2, the nocturnal secretion of TSH was subnormal before surgery. The alteration in thyroid function was reversible after the remission of hypercortisolism (mean time of recovery: 8 months), and the degree of central hypothyroidism was related to the duration of symptoms, morning and midnight serum cortisol, and urinary-free cortisol [27]. Interestingly, the HPT axis recovery time seemed to be inversely related to the severity of hypercortisolism, in which elevated urinary-free cortisol levels (≥1000 mcg/day) are a prognostic marker of longer recovery time. In addition, secondary hypogonadism was more frequent in CS patients affected by hypothyroidism [27]. This can be explained by an additional effect of hypercortisolemia on other hypothalamic–pituitary axes and by the fact that a normal hypothalamic–pituitary–gonadal axis function requires an intact HPT axis [27,32]. Cai et al. focused their retrospective evaluation only on ACTH-independent CS, studying a group of 94 patients affected by cortisol-secreting monolateral adrenal adenoma [33] and comparing them with a control group of patients with non-functioning adrenal incidentalomas. Reduced TSH levels during the active phase of the disease have been confirmed, but in contrast with previous studies, FT4 levels were lower also in ACTH-independent hypercortisolism when compared with controls. The normalization of both TSH and FT4 levels was observed after curative adrenalectomy [33].

In summary, most studies observed a reduction in TSH levels and an altered TSH secretion rhythm in patients with endogenous CS. On the contrary, data about the reduction of FT3 and FT4 levels are conflicting, which is probably due to the presence of heterogeneous groups of patients as well as to the different nature of the studies (retrospective vs. prospective), which makes comparison difficult. Overall, the changes in thyroid function seem to be reversed with successful treatment with a recovery time that is inversely related to the severity of hypercortisolism.

## 4. Exacerbation of Thyroid Disease after Surgery for Cushing’s Syndrome

In clinical experience, patients with endogenous Cushing’s syndrome have shown a remarkably high prevalence of primary thyroid disease [34]. A retrospective evaluation of 59 patients with CS found that 30.5% had goiter, 23.7% had primary subclinical hypothyroidism, and 8.4% had hyperthyroidism [34]. Moreover, the resolution of hypercortisolism seems to represent a trigger for the development of autoimmune disorders. In humans, the most frequently reported autoimmune diseases after the remission of CS are autoimmune thyroid diseases [35]. The first observations have been reported by Takasu et al., who described a small case series of patients with ACTH-independent CS, who underwent either unilateral [36] or bilateral [37] adrenalectomy and who developed autoimmune thyroid diseases. In those years, great interest developed about the immunoendocrine interactions and autoimmunity. It is reasonable to hypothesize that in patients with underlying autoimmune thyroid disease, the inappropriate cortisol secretion guarantees an immunologic tolerance masking the overt thyroid dysfunction, which develops only after the remission of hypercortisolism. It is well known that glucocorticoid excess exerts an inhibitory action on immune function in humans, as confirmed by the involution of lymphoid tissue and by the increased susceptibility to infections detected in patients with CS [35,38]. On the other hand, the exacerbation of several autoimmune diseases was reported both in adult and pediatric patients “cured” from hypercortisolism [35,39,40,41]. In his Editorial, McGregor commented on the three cases of CS previously described by Takasu, who developed after adrenalectomy a first phase of transient hyperthyroidism with the characteristics of a destructive thyroiditis, followed by hypothyroidism, and compared the immunological bases of this condition with that of post-partum thyroiditis [38].

Colao and coll. evaluated the prevalence of autoimmune thyroid diseases in 20 patients with CD and the possible association between previous thyroid disease (nodular goiter or positive thyroid autoantibodies during the active phase of hypercortisolism) and the subsequent development of autoimmune thyroid diseases during CD remission. A significantly higher prevalence of autoimmune thyroiditis (35%) was found in patients cured from CD than in patients with active CD and in controls. Furthermore, the development of autoimmune thyroiditis during the remission was significantly associated with the presence of a previous nodular goiter or positive thyroid autoantibodies titer during the active phase of the disease [30]. Other studies and case reports confirmed the increase in anti-thyroid antibodies titer after the resolution of hypercortisolism [42]. In the retrospective analysis of Niepomniszcze and coll. in 59 CS patients, the prevalence of positive anti-thyroid antibodies titers was 26.7% and 86.7% before and after surgery, respectively [34].

The onset and/or exacerbation of Graves’ disease after CS curative treatment has also been reported, but mostly by case reports [43,44,45,46,47]. It is reasonable to hypothesize the same pathogenetic mechanism and that, in predisposed patients, the autoimmune condition is in a “honeymoon” phase during active CS due to the increased immunologic tolerance induced by the steroid excess. In line with this, animal models employed to elucidate the impact of basal levels of adrenal gland-derived glucocorticoids on T cell activity showed that the withdrawal of adrenal glucocorticoids produces alterations in the thymocyte selection processes, increasing the risk to generate potentially self-reactive cells [48]. Moreover, since the onset of Graves’ disease and the risk of recurrences are associated with exposure to stressful events [49], it is possible to speculate that the sudden decrease in cortisol levels after successful treatment may represent itself as a stressor triggering the development of autoimmune hyperthyroidism.

Apart from autoimmune thyroid diseases, other conditions have been described after the successful treatment of Cushing’s syndrome. De Quervain’s thyroiditis (DQT) is a self-limiting inflammatory disorder of the thyroid gland due to a viral infection and an uncommon cause of thyrotoxicosis [50]. The occurrence of DQT has been reported in two patients after treatment of CS [51]. The first patient developed DQT symptoms (fever, neck pain, thyrotoxicosis) during medical treatment with pasireotide, while the second patient had the same symptoms after monolateral adrenalectomy for ACTH-independent CS. As reported by the authors, DQT, which is a steroids-responsive condition, occurred in both patients after the reduction of endogenous serum cortisol [51]. A possible physiopathological explanation is that the significant changes in glucocorticoids levels after the successful treatment of hypercortisolism may affect the susceptibility to infections through a modulation of the Th1/Th2 balance [52]. Another hypothesis is that the reduction of serum cortisol could affect the thyroid cells by inducing damage on vascular smooth muscle cells and on the endothelium [51].

The syndrome of inappropriate TSH secretion (SITS) is characterized by high concentrations of circulating thyroid hormones in the presence of non-suppressed TSH. After the exclusion of possible laboratory interferences, TSH-secreting pituitary adenoma (TSHoma) or resistance to thyroid hormones syndrome should be suspected. In 2013, Tamada et al. reported for the first time two cases of SITS after surgery for Cushing’s syndrome caused by the rapid decrease in cortisol levels and insufficient hydrocortisone replacement therapy [53]. Other causes of SITS were excluded. The authors also checked thyroid function in another seven patients who underwent successful surgery for CS and reported five cases of inappropriate TSH secretion, developing within 2 months after surgery [53]. Based on patients’ clinical courses, the rapid fall in glucocorticoid concentration after surgery for Cushing’s syndrome was probably the cause of SITS. As mentioned above, TSH levels increase after successful surgery for Cushing’s syndrome. The increase of thyroid hormones levels and the inability to adequate suppress TSH detected in SITS patients can be due to an impaired deiodinase activity. Type 2 deiodinase converts T4 to T3 in several tissues, including the hypothalamus and pituitary and contributes to the regulation of the HPT axis, regulating the local concentration of T3 [8]. D2 knockout animal models showed pituitary resistance to thyroid hormones [54], and defects in the synthesis of selenoproteins in humans cause the so-called “syndromes of reduced sensitivity to thyroid hormones” [8]. Tamada et al. supposed that a local reduction of D2 activity caused by adrenal insufficiency could decrease the local T3 concentration in the hypothalamus and pituitary and could induce SITS [53], but this hypothesis should be confirmed by more robust evidence. In the prospective study by Dogansen et al. (20 CD and 15 ACTH-independent CS), inappropriate TSH secretion syndrome has not been detected in any patient after surgery. The authors explained that these findings could be due to the adequate post-operative replacement treatment with prednisolone in their cohort [24].

## 5. Drugs Used for Cushing’s Syndrome and Their Effects on Thyroid Function

Regardless of its etiology, the treatment of choice for CS is represented by surgery: pituitary adenomectomy for CD, adrenalectomy for ACS, and removal of the ectopic ACTH-secreting tumor for EAS [55]. Medical therapies are used to control hypercortisolism before surgery or as a second-line option in patients in whom surgery is not curative or not possible, especially in ACTH-dependent CS. Three main drug categories are usually employed: pituitary-directed drugs, including pasireotide and cabergoline, aimed to reduce the ACTH production; steroidogenesis inhibitors, including ketoconazole, metyrapone, and mitotane; and glucocorticoid receptor (GR) antagonist, mainly represented by mifepristone.

### 5.1. Effects of ACTH Directed Drugs: Pasireotide and Cabergoline on HPT Axis Activity

Pituitary directed medical therapies are aimed to target the underlying cause of the disease, to reduce the ACTH secretion and inhibit tumor growth. The currently available pituitary-directed drugs include the SSTR ligand (SRL) pasireotide and the dopamine agonist (DA) cabergoline. Although pasireotide and cabergoline can be used for the biochemical control of the inappropriate TSH secretion in TSHomas [56,57], data regarding their effects on HPT axis activity in patients with CS are lacking and not well elucidated.

Pasireotide (SOM 230) is a “second generation” SSTR ligand (SRL) with relevant affinity for SSTR1, SSTR2, SSTR3, and particularly for SSTR5 [58] that is able to induce a significant decrease in ACTH secretion through the activation of SSTR2 and SSTR5, expressed on the pituitary corticotroph cells [59]. Pasireotide is currently the only pituitary-directed drug with an official license by the European Medical Agency (EMA) and the American Food and Drug Administration (FDA) for the treatment of CD in patients for whom surgery is not an option or in case of persistent disease after surgery [60]. It is tempting to speculate that due to its effect on SSTR2 and SSTR5 on pituitary thyrotropes, pasireotide would inhibit TSH secretion and result in central hypothyroidism. However, studies evaluating the effects of pasireotide on the hypothalamus–pituitary–thyroid axis are lacking. In primary cultures of rat pituicytes, pasireotide dose-dependently inhibited GH and TSH release, while in cultures of human fetal pituitary cells, pasireotide inhibited GH secretion but had no effect on TSH release [61]. In an above-mentioned case report [51], the use of pasireotide has been associated with the development of DQT, but more as the consequence of the reduction in serum cortisol levels rather than a direct drug-mediated effect.

To our knowledge, no drug-related effects on HPT axis have been reported in CD patients treated with cabergoline. In a small group of women with hyperprolactinemia treated with cabergoline, no variation in basal and TRH-stimulated TSH levels was found during medical treatment [62].

### 5.2. Effects of Steroidogenesis Inhibitors on HPT Axis Activity

The steroidogenesis inhibitors are adrenal-directed drugs that induce a decrease in cortisol secretion through the inhibition of specific enzyme pathways [60]. The drugs available to date include metyrapone, ketoconazole, and mitotane.

Metyrapone is an oral steroidogenesis inhibitor that acts on 11β-hydroxylase activity, blocking the conversion of 11-deoxycortisol to cortisol and of 11-deoxycorticosterone to corticosterone [63]. A study performed on a small group of healthy subjects (12) showed that metyrapone increased daytime mean TSH levels by 35%, with no change in nocturnal TSH secretion. There were no changes in TSH responses to TRH or in serum T3 or free T4 levels [64].

The anti-steroidogenic activity of ketoconazole, which has been worldwide used as an off-label treatment in the management of CS during the last 30 years [60], is based on the inhibition of several steroidogenesis enzymes (cholesterol side-chain cleavage complex, 17,20-lyase, 11β-hydroxylase, 17β-hydroxylase), inducing a reduction in glucocorticoid, mineralocorticoid, and adrenal androgens synthesis [63]. Furthermore, this drug also acts on ACTH secretion, suggesting a possible double effect in the treatment of CD patients [60]. Ketoconazole may exert anti-thyroid activity, which has been demonstrated by in vitro and in vivo. In vitro, ketoconazole was found to form a complex with iodine and to inhibit lactoperoxidase, while in vivo effects consisted in reduced circulating T4 levels and in increased thyroid gland weight in rats treated with ketoconazole [65]. These findings have been confirmed in similar animal models, showing decreased T3 and T4 levels, increased TSH, and changes in histology of the thyroid gland after ketoconazole treatment [66].

However, to our knowledge, no effects on thyroid function in CS patients treated with metyrapone or ketoconazole have been reported.

Mitotane is an adrenolytic drug that is currently used in the management of adrenocortical carcinoma but occasionally employed in the treatment of severe CS [67]. Mitotane effects on the HPT axis have been reported both in pre-clinical models and in clinical practice. In experimental models, the use of mitotane has been associated to an impaired TSH expression and TRH responsivity [68]. In clinical studies, a marked reduction in FT4 levels (inversely correlated with mitotane concentrations) has been reported, but interestingly, TSH did not change accordingly [69]. The explanation can be attributed to a possible “central hypothyroidism” or alternatively to an impairment of deiodinase activity with a change in FT4/FT3 ratio. Anyway, a thyroid function test should be performed during mitotane treatment, and some patients can require LT4 replacement therapy [70].

### 5.3. Effects of Glucocorticoid Receptors Antagonist Mifepristone on HPT Axis Activity

Mifepristone (RU486) is a progesterone receptor antagonist that has glucocorticoid receptor antagonist activity at high concentrations. In animal models, it has been demonstrated that hydrocortisone dose-dependently stimulated TSH-induced iodide uptake in porcine thyroid cells. Mifepristone, acting on the same intracellular cAMP pathways, inhibited the iodine uptake induced by hydrocortisone [71]. According to these findings, in humans, the use of mifepristone has been associated to alterations of thyroid function in CS patients. The effects of prolonged administration of mifepristone were studied in a small group of patients (4 females and 3 males) with unresectable meningioma, which is treated with mifepristone (200 mg/d) for 20 to 40 months. Serum samples were collected at monthly intervals. TSH values increased significantly, and the most pronounced increase was evident during the first 3 months of mifepristone treatment, but they remained within the normal range. Furthermore, a transient decrease in serum T4 has been detected after 2–3 months. Mifepristone concentrations were correlated with serum TSH and cortisol levels, suggesting that the alterations in the HPT and hypothalamus–pituitary–adrenal axes occurred in a concentration-dependent manner [72]. A clinical trial evaluating the safety and efficacy of mifepristone in endogenous CS found that eight of 42 patients had reversible increases in TSH during the treatment [73], which normalized six weeks after mifepristone discontinuation. Different hypotheses have been proposed to explain the effects of mifepristone on thyroid function, but they are not yet fully elucidated. The increased TSH secretion can be explained by the hypothalamic and pituitary GR inhibition [74]. Furthermore, an underlying thyroid disorder (autoimmune thyroid disease) can be masked by hypercortisolism and then can appear after the correction of hypercortisolism, as reported after successful surgery [36]. Recent consensus recommendations suggest that all patients should undergo a baseline thyroid function test before starting mifepristone treatment as well as to check thyroid function every 3 months or in the presence of any signs or symptoms consistent with abnormal thyroid function [74]. A case series of five patients affected by CD and central hypothyroidism showed that mifepristone treatment increased the levothyroxine requirement (median 1.83 times the initial dose of levothyroxine to achieve normal FT3 and FT4 levels). Altered intestinal absorption as well as increased inactivation of T4 related to deiodinase activity has been proposed as a possible mechanism explaining the increased requirement [75].

The effects of the drugs employed in CS treatment on the HPT axis are summarized in Table 1.

## 6. Conclusions

The effects of hypercortisolism on HPT axis activity are complex and involve different mechanisms. The most evident consequence of the inappropriate cortisol secretion on thyroid axis activity is a “central hypothyroidism”, which is due to the inhibitory effects of glucocorticoids on TRH and TSH secretion. Peripheral actions of thyroid hormones can be altered by an impaired function of deiodinases, which is related to the inappropriate cortisol secretion. However, the clinical interpretation and significance of dynamic changes in HPT function during active CS syndrome is still debated. The studies are mainly retrospective and included small and heterogeneous groups of patients. Furthermore, in the clinical practice, the possible effects of some drugs used for the control of hypercortisolism should be considered. Based on recent findings, the evaluation of thyroid function in CS patients both during active disease and after the remission of hypercortisolism represents a reasonable approach. In fact, some dynamic changes in HPT axis activity are recovered after successful surgery. Furthermore, the increased prevalence of autoimmune thyroid disease observed after hypercortisolism remission must be considered. Probably, in patients with underlying autoimmune thyroid disease, the inappropriate cortisol secretion guarantees an immunologic tolerance masking the overt thyroid dysfunction, which develops after the cure of CS.

## Figures and Tables

**Table 1:** Effects of CS drugs on thyroid function.

Drug	Test Results			Condition Mimicked
	TSH	FT4	FT3	
**Pasireotide**	NR	NR	NR	-
**Cabergoline**	NR	NR	NR	-
**Metyrapone**	35% increase compared to baseline *	Normal	Normal	-
**Ketoconazole**	NR	NR	NR	-
**Mitotane**	Normal	Low	Normal	Central hypothyroidism
**Mifepristone**	High	Normal	NR	Subclinical hypothyroidism

* reported in healthy subjects. No alterations reported in CS patients. NR = not reported.
